# A Retrospective Study of Climate Change Affecting Dengue: Evidences, Challenges and Future Directions

**DOI:** 10.3389/fpubh.2022.884645

**Published:** 2022-05-27

**Authors:** Surbhi Bhatia, Dhruvisha Bansal, Seema Patil, Sharnil Pandya, Qazi Mudassar Ilyas, Sajida Imran

**Affiliations:** ^1^Department of Information Systems, College of Computer Sciences and Information Technology, King Faisal University, Al-Ahsa, Saudi Arabia; ^2^Symbiosis Institute of Technology, Symbiosis International (Deemed) University, Pune, India; ^3^Department of Computer Engineering, College of Computer Sciences and Information Technology, King Faisal University, Al-Ahsa, Saudi Arabia

**Keywords:** predictive models, machine learning, surveillance system, dengue, climatic factors

## Abstract

Climate change is unexpected weather patterns that can create an alarming situation. Due to climate change, various sectors are affected, and one of the sectors is healthcare. As a result of climate change, the geographic range of several vector-borne human infectious diseases will expand. Currently, dengue is taking its toll, and climate change is one of the key reasons contributing to the intensification of dengue disease transmission. The most important climatic factors linked to dengue transmission are temperature, rainfall, and relative humidity. The present study carries out a systematic literature review on the surveillance system to predict dengue outbreaks based on Machine Learning modeling techniques. The systematic literature review discusses the methodology and objectives, the number of studies carried out in different regions and periods, the association between climatic factors and the increase in positive dengue cases. This study also includes a detailed investigation of meteorological data, the dengue positive patient data, and the pre-processing techniques used for data cleaning. Furthermore, correlation techniques in several studies to determine the relationship between dengue incidence and meteorological parameters and machine learning models for predictive analysis are discussed. In the future direction for creating a dengue surveillance system, several research challenges and limitations of current work are discussed.

## 1. Introduction

**Figure 1:**
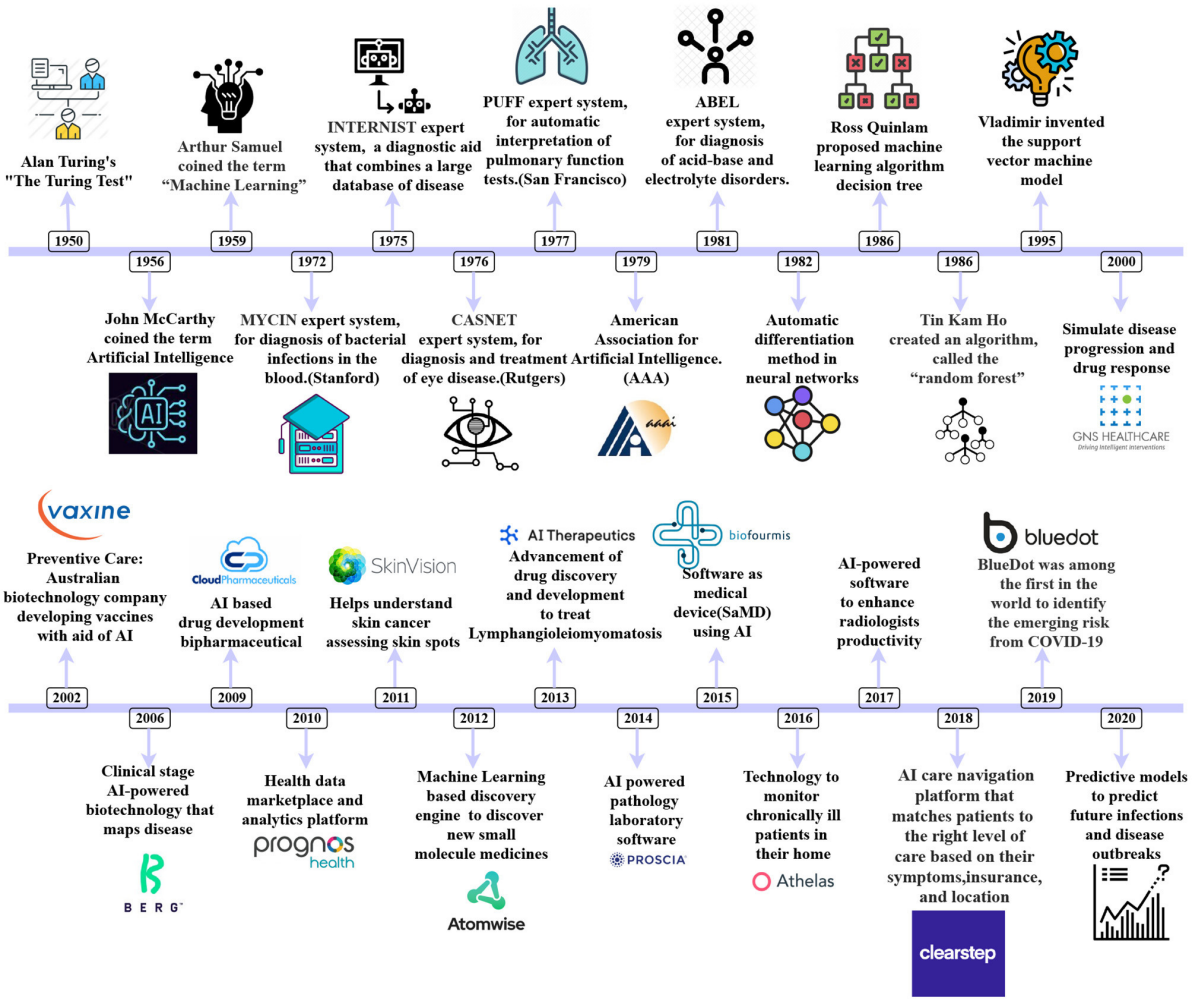
A chronological representation of Artificial Intelligence in Healthcare from 1952 to 2020.

**Figure 2:**
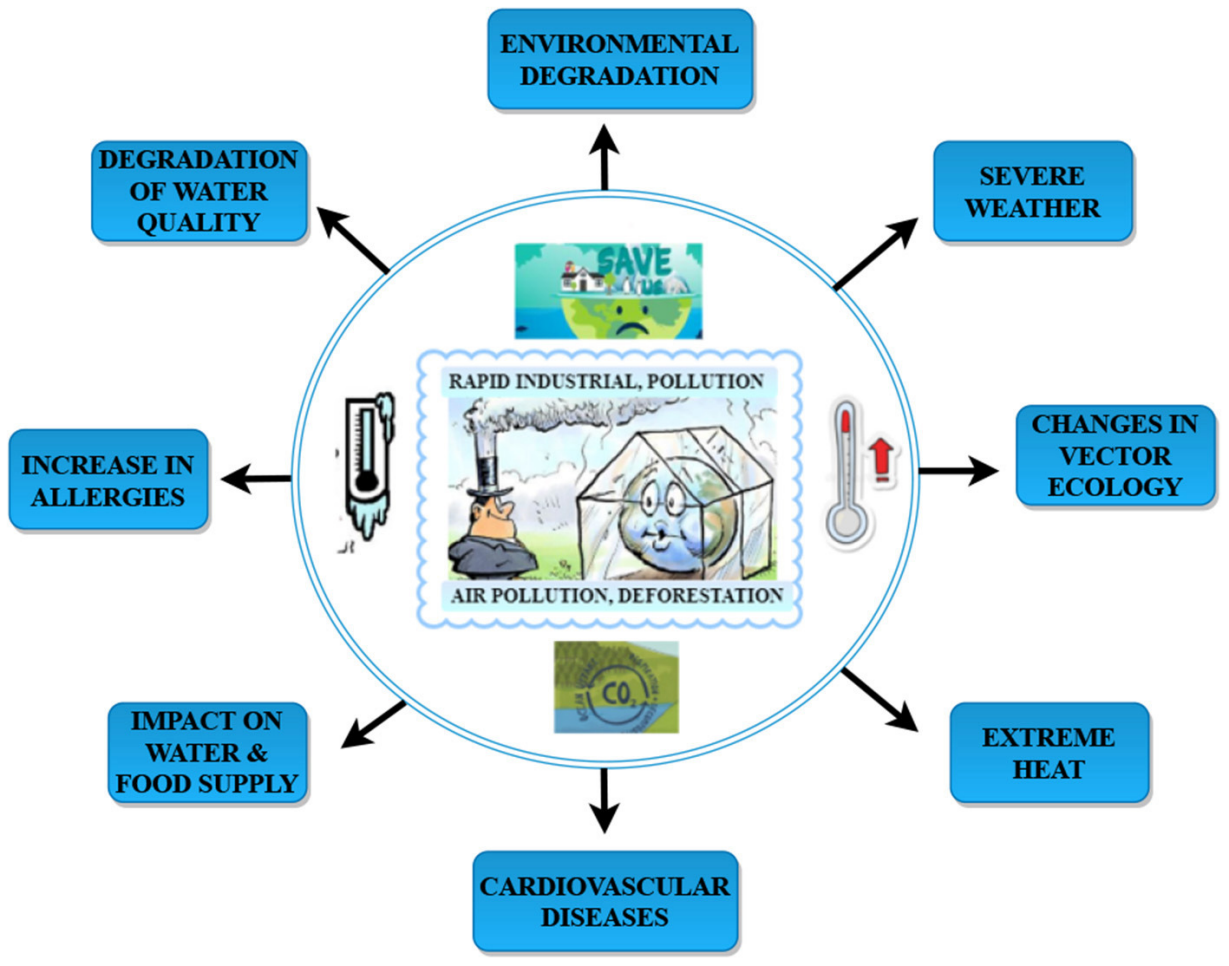
Causes and consequences of climate change.

Artificial Intelligence is a field in which a machine exhibits intelligence by learning about itself. Artificial Intelligence may employ various approaches and algorithms to comprehend human intellect, but it is not limited. Machine Learning is a branch of AI that deals with techniques that can learn from experience on their own (1). In automated decision-making and predictive analytics, AI plays a crucial role (2). Methods based on various kinds of AI are already being used in a variety of climate change and environmental monitoring research sectors (3). Machine Learning and Deep Learning are used in education, finance, healthcare, agriculture, and many more. The application of Machine Learning in healthcare demonstrated encouraging results, instilling trust in the sector (4). There are varied reasons for using artificial intelligence in healthcare. Primarily, it helps to manage the clinical records of patients and aids in providing personalized treatment and medicines, early identification of disease, providing predictive analytics, which could help health officials take preventive measures in advance before the outbreak of any disease (5). Artificial Intelligence goes back to the too early 1990s and today has evolved into a burgeoning field with a significant contribution in healthcare (6) through various modes like drug and discovery, personalized treatment and medicine, preventive measures, a health assistant chatbot, clinical trial and researches, diagnosing and pathology, analytics, image processing and prediction of an outbreak. Artificial Intelligence is a boon, and Figure 1 demonstrates the evolution of AI in healthcare throughout 1950–2020. Climate change is long-term changes in temperature and weather patterns.These variations might be attributed to natural causes such as solar cycle oscillations. Due to the use of fossil fuels such as coal, oil, and gas, human activities have been the leading cause of climate change since the 1800s (7). Greenhouse gas emissions from fossil fuel combustion act as a blanket, trapping the sun's heat and boosting global temperatures. The amount of greenhouse gases in the atmosphere is at its highest level in 2 million years. Artificial Intelligence is a field in which a machine exhibits intelligence by learning about itself. Artificial Intelligence may employ various approaches and algorithms to comprehend human intellect, but it is not limited. Machine Learning is a branch of AI that deals with techniques that can learn from experience on their own. In automated decision-making and predictive analytics, AI plays a crucial role. Methods based on various kinds of AI are already being used in a variety of climate change and environmental monitoring research sectors. Machine Learning and Deep Learning are used in education, finance, healthcare, agriculture, and many more. The application of Machine Learning in healthcare demonstrated encouraging results, instilling trust in the sector (8). There are varied reasons for using artificial intelligence in healthcare. Primarily, it helps to manage the clinical records of patients and aids in providing personalized treatment and medicines, early identification of disease, providing predictive analytics, which could help health officials take preventive measures in advance before the outbreak of any disease. Artificial Intelligence goes back to the too early 1990s and today has evolved into a burgeoning field with a significant contribution in healthcare through various modes like drug and discovery, personalized treatment and medicine, preventive measures, a health assistant chatbot, clinical trial and researches, diagnosing and pathology, analytics, image processing and prediction of an outbreak. Artificial Intelligence is a boon, and Figure 1 demonstrates the evolution of AI in healthcare throughout 1950–2020. Climate change is long-term changes in temperature and weather patterns.These variations might be attributed to natural causes such as solar cycle oscillations. Due to the use of fossil fuels such as coal, oil, and gas, human activities have been the leading cause of climate change since the 1800s. Greenhouse gas emissions from fossil fuel combustion act as a blanket, trapping the sun's heat and boosting global temperatures. The amount of greenhouse gases in the atmosphere is at its highest level in 2 million years. Emissions are steadily increasing. Since the late 1800s, the Earth has warmed by around 1.1°C. The previous 10 years (2011–2020) have been the hottest on record. Figure 2 explains the causes of climate change and the effects due to those variations. Vector-borne illness is a severe public health threat in underdeveloped nations that is only becoming worse. Temperatures in the air and water, precipitation patterns, severe rainfall events, and seasonal changes are all known to impact disease transmission (9). At the moment, dengue (10) is spreading widely after COVID-19; dengue is a vector-borne disease that spreads through an infected female mosquito of species Aedes aegypti (11) and is one of the most common diseases in more than 100 countries (12). Aedes aegypti can be found in urban and suburban settings with high human population density and housing density. This species is endophilic, which means it seeks shelter inside structures. As a result, the intradomicile is more typically seen than the peridomicile. Tires, cans, bottles, pots, vats, brass, swimming pools, and abandoned aquariums are frequent breeding habitats filled with rainwater or household water.

**Figure 3:**
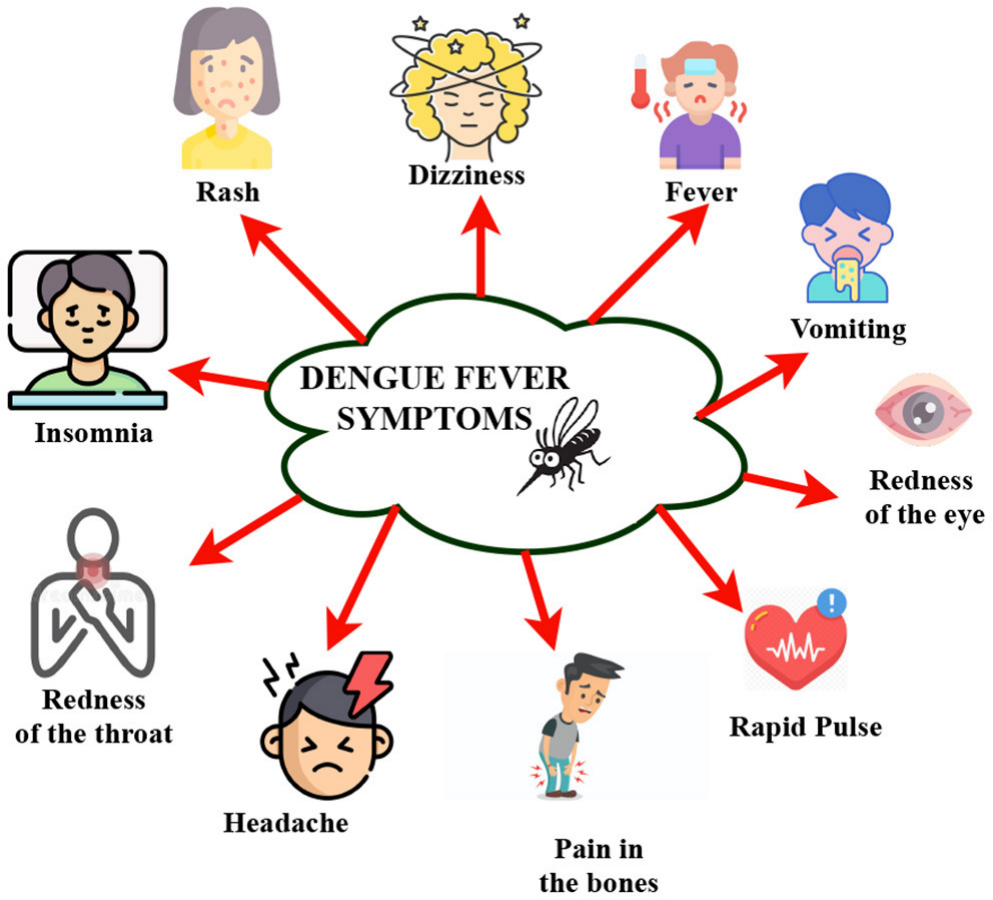
Symptoms of dengue.

**Figure 4:**
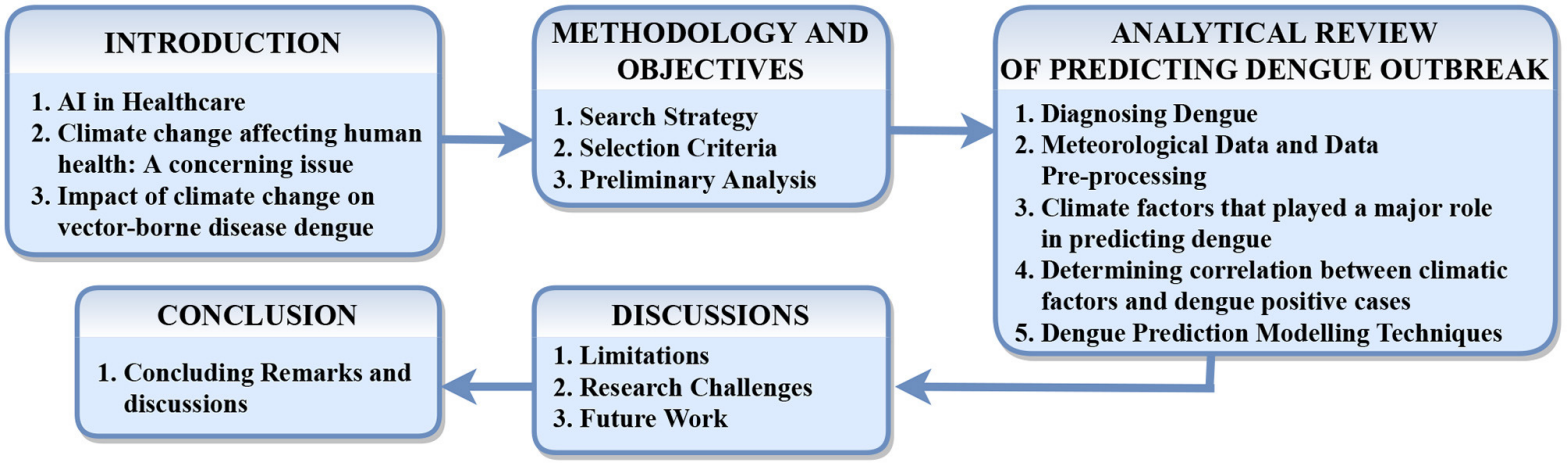
Roadmap of the conducted review.

These mosquitos carry the Chikungunya, yellow fever, and Zika viruses. Dengue fever is widespread in the tropics, with different risk levels based on rainfall, temperature, relative humidity, and unplanned fast urbanization. Dengue fever may be fatal if it is not treated appropriately. It was first discovered during dengue epidemics in the Philippines and Thailand in the 1950s. Most Asian and Latin American nations now have severe dengue fever, a leading cause of hospitalization and mortality among children and adults. A Flaviviridae virus causes dengue with four unique but closely related serotypes (DENV-1, DENV-2, DENV-3, DENV-4), and the fifth serotype of dengue has been detected in the Malaysian state of Sarawak (13). Dengue fever is caused by DENV infection in our bodies. Figure 3 depicts the common symptoms of dengue. Dengue fever causes severe joint and muscle pain, enlarged lymph nodes, headaches, fever, exhaustion, and a rash. Dengue fever is characterized by non-specific flu-like symptoms such as chills, appetite loss, lethargy, and low backache. According to a recent WHO research, dengue fever grew in several countries in 2020, including Bangladesh, Brazil, the Cook Islands, Ecuador, India, Indonesia, the Maldives, Mauritania, Mayotte, Nepal, Singapore, Sri Lanka, Sudan, Thailand, Timor-Leste, and Yemen. In 2019, dengue fever cases reached an all-time high in the world. Dengue fever is slowly gaining ground in 2021, with instances documented in several nations. Around 40 per cent of the world's population lives in areas with a high risk of dengue fever, such as tropical and subtropical climates. Dengue fever has become much more common in recent decades worldwide. According to one estimate, 390 million dengue virus infections occur each year (12). Emissions are steadily increasing. Since the late 1800s, the Earth has warmed by around 1.1°C. The previous 10 years (2011–2020) have been the hottest on record (9). Figure 2 explains the causes of climate change and the effects due to those variations. Vector-borne illness is a severe public health threat in underdeveloped nations that is only becoming worse. Temperatures in the air and water, precipitation patterns, severe rainfall events, and seasonal changes are all known to impact disease transmission (10). At the moment, dengue (11) is spreading widely after COVID-19; dengue is a vector-borne disease that spreads through an infected female mosquito of species Aedes aegypti (12) and is one of the most common diseases in more than 100 countries (14). Aedes aegypti can be found in urban and suburban settings with high human population density and housing density. This species is endophilic, which means it seeks shelter inside structures. As a result, the intradomicile is more typically seen than the peridomicile. Tires, cans, bottles, pots, vats, brass, swimming pools, and abandoned aquariums are frequent breeding habitats filled with rainwater or household water (13). These mosquitos carry the Chikungunya, yellow fever, and Zika viruses. Dengue fever is widespread in the tropics, with different risk levels based on rainfall, temperature, relative humidity, and unplanned fast urbanization. Dengue fever may be fatal if it is not treated appropriately. It was first discovered during dengue epidemics in the Philippines and Thailand in the 1950s. Most Asian and Latin American nations now have severe dengue fever, a leading cause of hospitalization and mortality among children and adults. A Flaviviridae virus causes dengue with four unique but closely related serotypes (DENV-1, DENV-2, DENV-3, DENV-4), and the fifth serotype of dengue has been detected in the Malaysian state of Sarawak (15). Dengue fever is caused by DENV infection in our bodies. Dengue fever causes severe joint and muscle pain, enlarged lymph nodes, headaches, fever, exhaustion, and a rash. Dengue fever is characterized by non-specific flu-like symptoms such as chills, appetite loss, lethargy, and low backache. According to a recent WHO research, dengue fever grew in several countries in 2020, including Bangladesh, Brazil (16), the Cook Islands, Ecuador, India (17), Indonesia (18), the Maldives, Mauritania, Mayotte, Nepal, Singapore (19), Sri Lanka, Sudan, Thailand (20), Timor-Leste, and Yemen. In 2019, dengue fever cases reached an all-time high in the world. Dengue fever is slowly gaining ground in 2021, with instances documented in several nations (14). Around 40 per cent of the world's population lives in areas with a high risk of dengue fever, such as tropical and sub-tropical climates. Dengue fever has become much more common in recent decades worldwide. According to one estimate, 390 million dengue virus infections occur each year (14). Dengue fever will affect 60 per cent of the world's population by 2080, according to researchers who blame climate change for the disease's spread. Climate change is widely cited as a contributing factor in the rapid spread of pandemic disease (21). Climate change is frequently identified by WHO, national, and international health authorities as one of the primary causes of the global spread of dengue fever and other Aedes-transmitted viral infections. According to the World Health Organization, dengue fever has become increasingly widespread internationally in recent decades (WHO). According to the World Health Organization, dengue fever affects 50–100 million people globally each year. As a result, it is vital to anticipate dengue epidemics. The accuracy of dengue epidemic prediction is currently a problem that must be addressed. The role of climate variables in predicting dengue outbreaks has been studied in a small number of studies. Tropical countries are the hardest hit because of their environmental, climatic, and socioeconomic characteristics (22). The weather has an impact on the vector-borne illness dengue's temporal and geographical spread. As a result, rainfall and ambient temperature are referred to as macro factors that influence dengue since they directly impact Aedes aegypti population density, which varies seasonally based on these important variables. Its population density tends to decline drastically during less precipitation and lower average temperatures in regions with a tropical or subtropical climate. Still, it grows consistently in locations with a tropical or subtropical environment (23). As the vector-weather association is as significant as the vector-human interaction, studies of climatic variables can have a better understanding of epidemic seasonality and the ability to anticipate it (24). In recent years, epidemiological research has focused on developing mathematical and statistical models based on weather parameters to explain the dynamics of dengue fever incidence. Its main goal was to find models with promising future predicting the potential of dengue incidence to help public health officials. Several researchers investigate the link between climate variables and dengue fever, frequently employing time-series analyses to characterize temporal trends, uncover patterns, and even make forecasts. Bhatt et al. (21) Many parameters, such as entomological, epidemiological, and geographic characteristics, can help predict the dengue outbreak effectively. Artificial intelligence has been employed in healthcare for a long time. This review study discusses the studies undertaken until now. Figure 4 represents road-map of the present study. It investigates several other vulnerable groups that will be useful in predicting positive dengue cases and eventually help prevent the outbreak and take preventive measures at an early stage. The present study is structured as follows: Section 2 describes the methodology and objectives of the survey carried out; Section 3 provides a detailed analytical review of the papers; Section 4 discusses the challenges and future work, and Section 5 concludes the present study.

## 2. Methodology and Objectives

To carry out the systematic literature review of dengue prediction models, process was followed which included i) establishing research questions, ii) developing a search strategy to narrow down the results, iii) selecting papers using eligibility criteria and iv) analyzing to extract strengths, weaknesses, and difficulties to overcome from articles. The main objectives of this systematic literature review are i) To gather and describe dengue machine learning models for dengue surveillance systems and ii) To illustrate the obstacles that future dengue modeling studies will face.

### 2.1. Search Strategy

Various digital libraries like Taylor and Francis, Google Scholar, MDPI, IEEE Xplorer, ScienceDirect, Pubmed, BMC and PLOS were used. The inclusion criteria were articles from January 2017 to August 2021 related to various dengue prediction models based on explanatory variables. The requirements for discarding the publications were personal opinions, unpublished works, posters, short articles and conference papers. The selection procedure included choosing themes by scrutinizing their title, keywords and abstract to eliminate the results that were not related. Further, to investigate more relevant papers, we examined summaries to determine if we could select the article or not.

### 2.2. Preliminary Analysis

**Figure 5:**
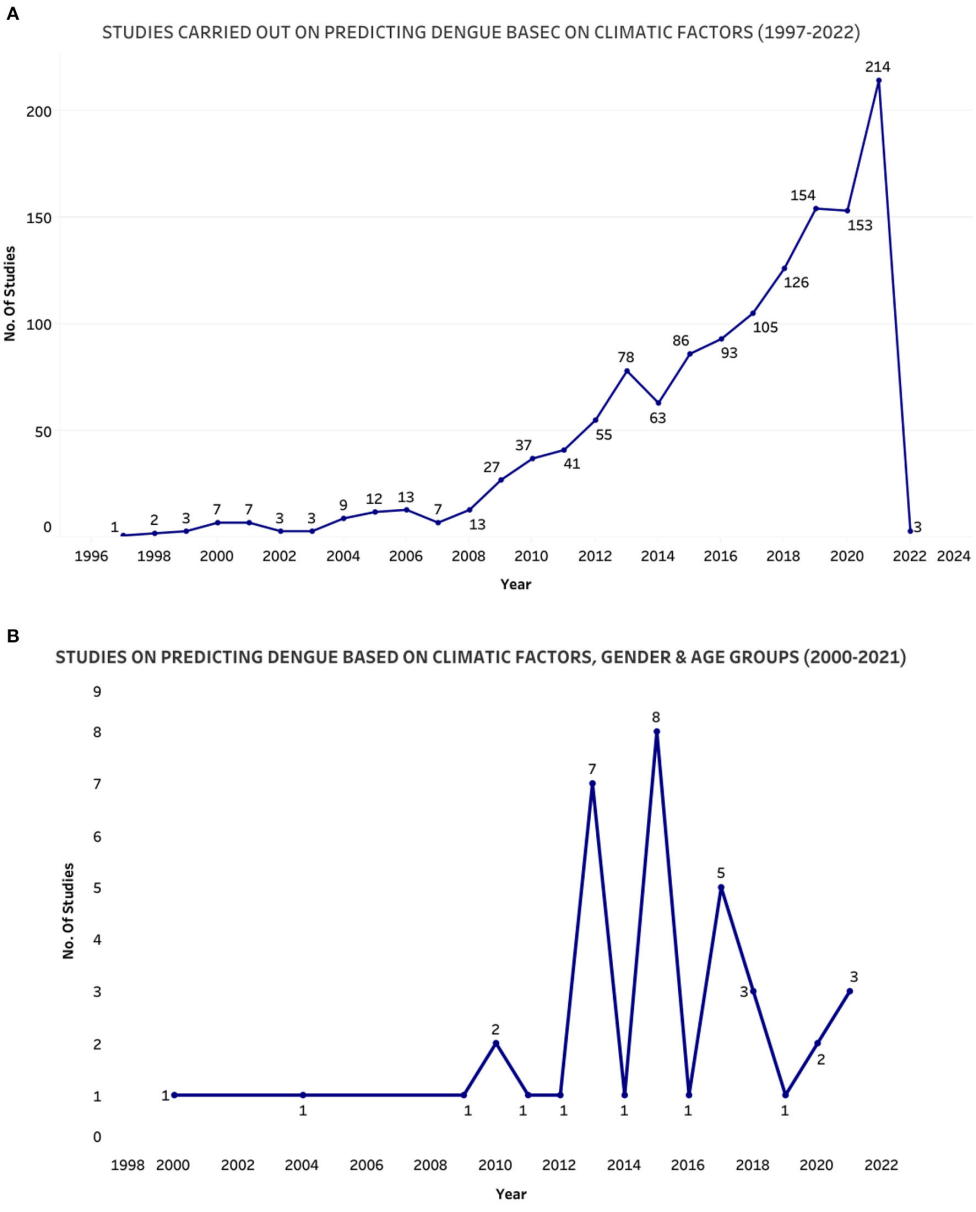
No. of studies in different regions and time-period of scopus database: **(A)** No. of studies carried out on predicting dengue based on climatic factors for the time period 1997–2022 and **(B)** No. of studies carried out on predicting dengue based on climatic factors, age group and gender for the time-period 2000–2021.

**Figure 6:**
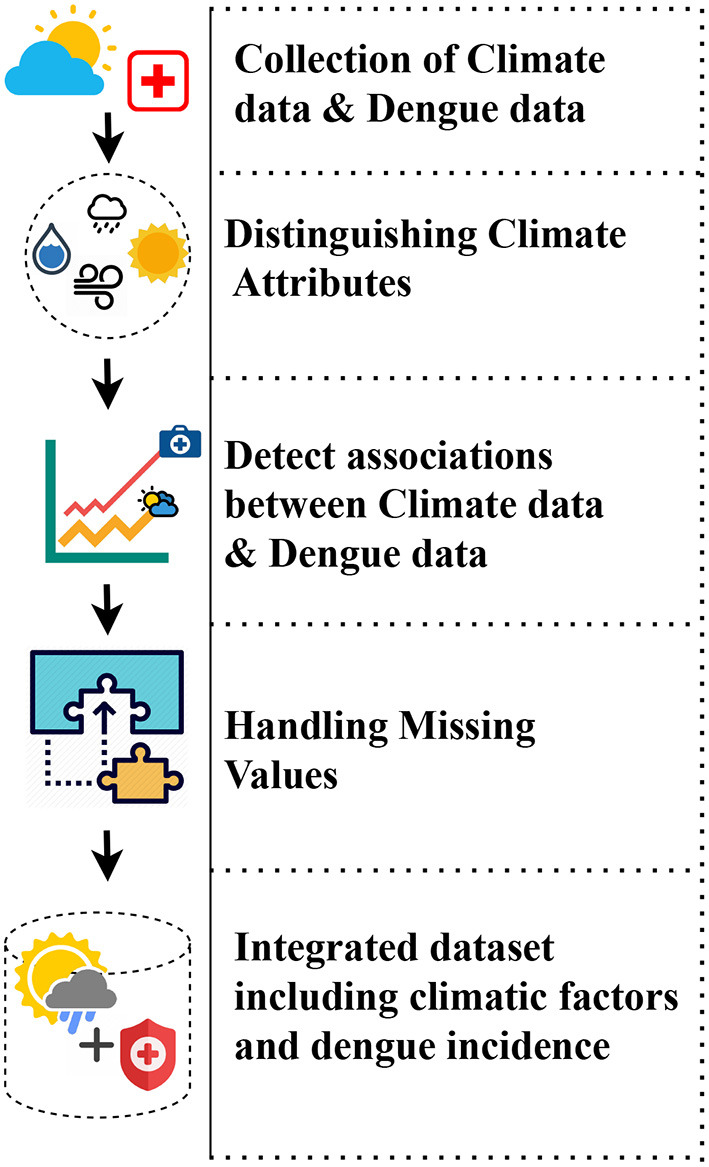
Data pre-processing stages.

To analyze the amount of research carried out, we analyzed Scopus data using keywords dengue, predicting, climatic factors, age and gender. Figure 5A were graphically represented using the query “[(predicting dengue) and (climatic factors and dengue)]” which fetched 1,315 documents, and Figure 5B were graphically represented using the query [(predicting dengue) AND (climatic factors and dengue) and (age group and gender)] which fetched only 38 documents. Therefore, we can deduce that studies on dengue prediction based on climatic factors, age group and gender has not been done by many researchers, and we can explore this field. As it is not carried out in many regions, there is a scope to examine countries/territories where this study has not been carried out. The analytic review that we carried out had the highest number of studies were from India, Brazil and Malaysia. Figure 6 displays the number of studies done in different countries and republics. As stated by the WHO report, these countries belong to tropical and sub-tropical regions that are more prone to dengue. Various explanatory variables are presently used for dengue modeling, categorized according to their characteristics and method of collection. The variables used were clinical, economic, laboratory and climatic factors. In general, climatic factors mainly were used. According to this review, vulnerable groups like age group and gender were least for dengue outbreak prediction as data about patients is not that easily available.

## 3. Analytical Review of Predicting Dengue Outbreak

Forty articles of period 2019–2021 were reviewed and analyzed to understand the dengue prediction models and how different factors like meteorological conditions, laboratory tests, symptoms, demographic features were dependent variables for the rise of dengue cases.

### 3.1. Diagnosing Dengue

Identifying the dengue infection, viral antigens, viral RNA, and antibodies against the virus in the patient's blood or tissues are among the laboratory procedures used to diagnose dengue. Only 4–5 days after symptoms can the virus be discovered in the blood (25). Dengue can be diagnosed by isolating the virus, viral RNA, and viral protein during the early stages of the disease. Identifying antibodies IgM and IgG in an infected person's blood is an indirect approach to diagnosing dengue fever. This approach is widely used to identify dengue fever in its latter stages after virus levels have dropped. Doctors can discover antibodies to dengue 5 days after the onset of symptoms in most individuals, and IgG can be detected for months or even years after infection. IgM levels are very high after a primary (initial) dengue infection, but they are decreased with subsequent infection. During a subsequent infection, IgG levels increase. As a result, doctors can use IgM and IgG concentrations to determine whether a patient has a primary or secondary dengue infection (26). Therefore, in most of the data, IgG and IgM and levels are specified for easy detection and also it can aid in creating the laboratory dengue surveillance system (27).

### 3.2. Meteorological Data and Data Pre-processing

Meteorological data represents the weather conditions like relative humidity, precipitation, minimum temperature, maximum temperature, air pressure, wind speed and many other parameters for location and time. These are massive data collected and later utilized to record the highest and lowest climatic events for various reasons like weather forecasting to identify seasonal change and public health. There are various sources through which we can obtain data. A brief description of the explanatory variables, data source, region and period is presented in the Table 1 used in different studies. Data Pre-processing is a crucial stage after data collection. This step enhances the model's performance, to which they will further provide the data for training and testing. Dengue incidence data collected contains information that might not be required, and there might be missing values. To handle these shortcomings, data pre-processing is a significant step. Figure 6 shows the steps followed in information pre-processing. The major concern for reviewers is missing data and data not being available completely (28). Jorge et al. (29) developed a three-stage to take the missing values in the dataset wherein first stage parameters with more than 20 per cent of missing values were eliminated. At the next stage, they discarded cases with a non-response rate higher than 80 per cent. There are no particular rules for selecting the proper imputation of missing variables. It relies on the dataset type, non-response type, pattern of loss of response, research aims, specific population features, general study organization characteristics, or available software. Given the data entry procedure's features and the dataset's epidemiological nature, they concluded that imputing the mean of the valid neighboring data to the missing values was the best option at the third stage (30).

**Table 1:** Detailed representation of explanatory variables and data sources of different studies.

**References**	**Region**	**Period**	**Explanatory variables/data source**
Leandro et al. (15)	Brazil	2007–16	Dengue Data: State Department of Health of the State of Rio de Janeiro Temperature: Moderate Resolution Imaging Spectroradiometer Rainfall : Tropical Rainfall Measuring Mission
Joseph et al. (27)	Philippines	2014–18	Age and Gender of patient: Philippine Epidemiological Bureau (Department of Health)
Daniel et al. (39)	Mexico	2010–14	Dengue Incidence Count/Ministry of Health of the State Government of San Luis Potosí Environmental (Temperature, Rainfall, Relative Humidity and Elevation above sea level): INEGI (National Institute of Statistics and Geography - Mexico) Proximity (Distance to water bodies, vegetation and roads): UGSG Social (Population, Piped Water Index, Drainage Index, Human Development Index): INEGI
Jian Cheng et al. (49)	China	2006–15	Daily number of dengue cases: China Centre for Disease Control and Prevention Temperature, Relative Humidity and Rainfall: National Meteorological Information Center
HaorongMeng et al. (50)	China	2006–18	Dengue Case Count: Chinese National Notifiable Infectious Disease Reporting Information System Temperature and Precipitation: China Meteorological Data Service Center
Kumar Shashvat et al. (51)	India	2014–17	Dengue Cases: Integrated Disease Surveillance Programme, Government of India. Rainfall and Relative Humidity: Indiastat.com
Sourabh Bal et al. (38)	India	2005–16	Records of dengue cases/Directorate of Health Services, Government of West Bengal Temperature, Relative Humidity and Rainfall: IMD
Pi Guo et al. (52)	China	2011–14	Dengue case count: China National Notifiable Disease Surveillance System Temperature, Relative Humidity and Rainfall: China Meteorological Data Sharing Service System
Wei Wu et al. (53)	China	2003–14	Dengue Cases: National Notifiable Infectious Disease Reporting Information System (NIDRIS) Temperature, Relative Humidity and Rainfall: WorldClim Population Density, Road Density, Land use and cover: RESDC / GeoSOS
Sabrina IslamI et al. (28)	Bangladesh	2002–13	Dengue Case Data: Directorate General of Health and Services Temperature, Relative Humidity and Rainfall: Bangladesh Meteorological Department
Teerawad Sriklin et al. (32)	Thailand	2015–19	Dengue Fever Cases: Bureau of Epidemiology, Ministry of Public Health Temperature, Rainfall, Air Pressure and relative humidity: Meteorological Department of Southern Thailand
Gayan et al. (54)	Sri Lanka	2005–17	Dengue Incidence Data: Regional Epidemiology Unit Temperature, Relative Humidity and Rainfall: Department of Meteorology, Colombo
Felestin et al. (55)	Malaysia	2010–13	Dengue Fever Confirmed Cases: Ministry of Health Malaysia (MOH) portal Temperature, Rainfall and Relative Humidity: Malaysian Meteorological Department

### 3.3. Climate Factors That Played a Major Role in Predicting Dengue

**Figure 7:**
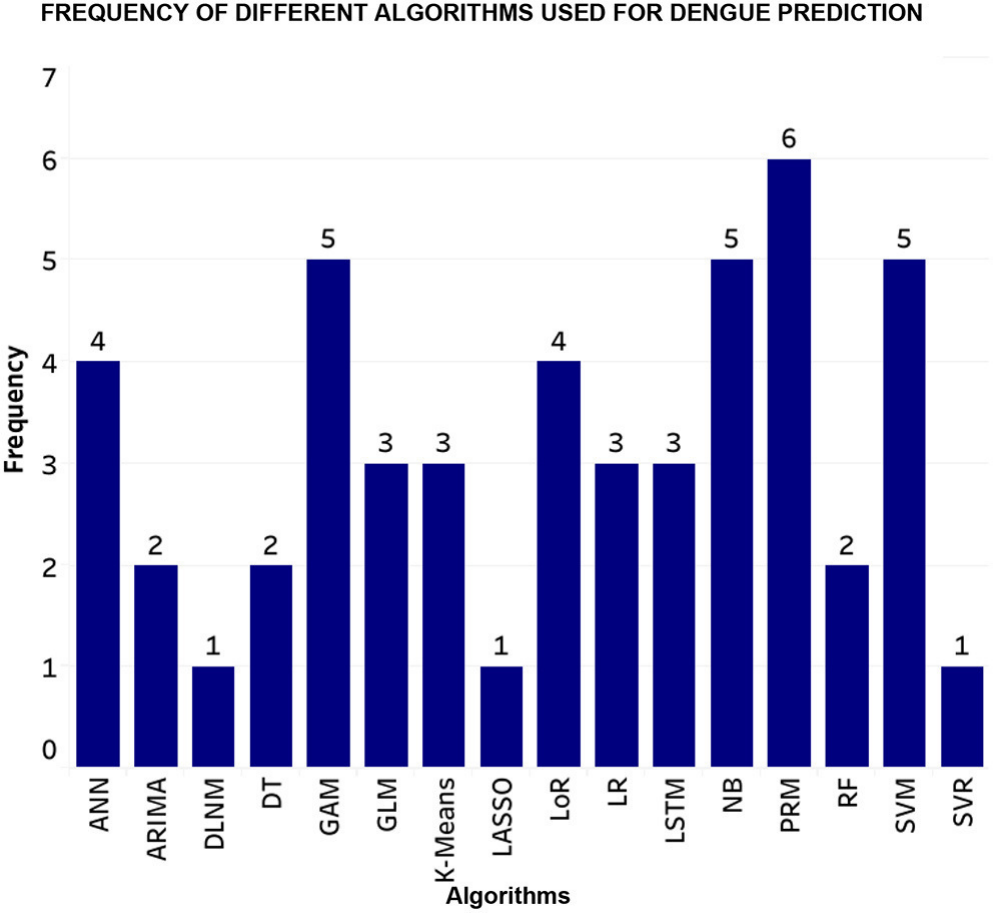
Frequency of machine learning techniques used for dengue incidence prediction (SVM, Support Vector Machine; NB, Naive Bayes; DLNM, Distributed Lag Non-Linear Model; PRM, Poisson Regression Model; LSTM, Long Short Term Memory, ANN, Artificial Neural Network; GLM, Generalized Linear Model; GAM, Generalized Additive Model; LoR, Logistic Regression; LR, Linear Regression; RF, Random Forest; DT, Decision Tree; SVR, Support Vector Regressor).

According to many studies, certain meteorological factors like temperature, rainfall, humidity, wind speed, air pressure and vegetation index displayed a positive correlation with the rise in dengue incidence. Hence, it helps us narrow down to a few concrete factors that can be used in further research to create dengue surveillance systems for different vulnerable groups. Figure 7 displays the percentage and combination of climatic factors that have been used in review papers, where temperature, rainfall and humidity account for 49 per cent. Therefore, other least used elements that show some relation with dengue incidence can be used for efficient prediction.

#### 3.3.1. Temperature

The most critical weather component affecting mosquito vector growth and dispersion and a potential predictor of dengue outbreak is temperature (31). The density of the mosquito population, biting rates, gonotrophic cycle lengths, and vector size are all affected by temperature during the mosquito's incubation life cycle and behavior. The findings of Teerawad Sriklin et al. study.'s back with prior research suggest that severe temperatures alter the development of dengue vectors. As a result, the temperature has an impact on vectorial efficiency as well as the probability of an epidemic. Given the link between temperature and dengue cases, the predicted temperature change could worsen dengue transmission (28, 32).

#### 3.3.2. Rainfall

Dengue transmission was found to be linked to rain and wet days. Rainfall has been identified as a role in the spread of dengue fever. Mosquitoes spend their whole life cycle in water before hatching into adult mosquitoes. Increased rainfall may create new habitat for larvae and vectors and boost adult survival (33).

#### 3.3.3. Humidity

Despite widespread interest in the relationship among climatic conditions and positive dengue cases prevalence among researchers, research on relative humidity as a critical climatic component has been limited. Furthermore, the findings of the few study were equivocal and contradictory. They showed relative humidity as the most crucial predictor in an Indonesian investigation of dengue outbreaks in that nation, with a 3–4-month lag time. According to this study, low humidity in September and October is generally followed by a dengue outbreak early the following year. As a result, it's highly likely that if seasonal circumstances vary as a result of climate change, seasonal dengue outbreaks will shift as well (34).

#### 3.3.4. Geographic Factors

The influence of residential regions on mosquito incidence became more evident under certain environmental circumstances, such as lower precipitation. According to research, mosquito incidence is very susceptible to high residential sites with a more significant density of residential streets. Roads feature drainage components that collect surface water runoff and release it in appropriate areas to avoid inland floods. However, concrete structures encroaching on channels or garbage filling them can often undermine adequate drainage in residential areas. These obstructions can prevent complete water flow, resulting in water accumulation places that can provide favorable habitat for Aedes aegypti. A growing body of research suggests a link between drainage and the presence of Aedes aegypti (28).

### 3.4. Determining Correlation Between Climatic Factors and Dengue Positive Cases

Correlation is a statistical method for deciding the degree to which two parameters are related, using correlation coefficients. Correlation coefficients are used to determine the strength of a linear relationship between two parameters. Correlation coefficient can be used to determine if a climatic element is positively or adversely associated with the increase of dengue fever cases (35). Various correlation strategies have been utilized in various research, and they have proven to help remove the climatic factors that were not very significant. The Pearson's correlation coefficient determines the statistical relationship between two variables. It is based on the covariance method. It is widely acknowledged as the most effective approach for determining the connection between two variables of interest. It offers information on the amount and direction of the link, or correlation, between the two variables. A non-parametric test called Spearman rank correlation predicts the degree of relationship among two variables. Pearson Correlation Coefficient (36) and Spearman's Rank Correlation Test (37) have been widely used. Sourabh Bal et al. carried out their study of dengue occurrence based on climatic factors for the region Kolkata, India, using auto-correlation coefficient and partial auto-correlation coefficient values. An auto-correlation analysis is used to examine dengue cases affected by prior instances. In addition, the Pearson correlation was used to assess for collinearity between the various climate variables (38). In the study carried out by Daniel Sánchez-Hernández et al. carried out correlation analysis to inspect they built the relationships of proximity, environmental, social factors and location about the occurrence of dengue, a multivariable logistic regression model. A logistic curve may be produced by graphing the connection between the explanatory and predicted variables, which will help us evaluate the correlation strength (39). Various correlation techniques have been according to the review Pearson, and spearman's correlation test were widely used and effective in most of the studies.

### 3.5. Dengue Prediction Modeling Techniques

**Figure 8:**
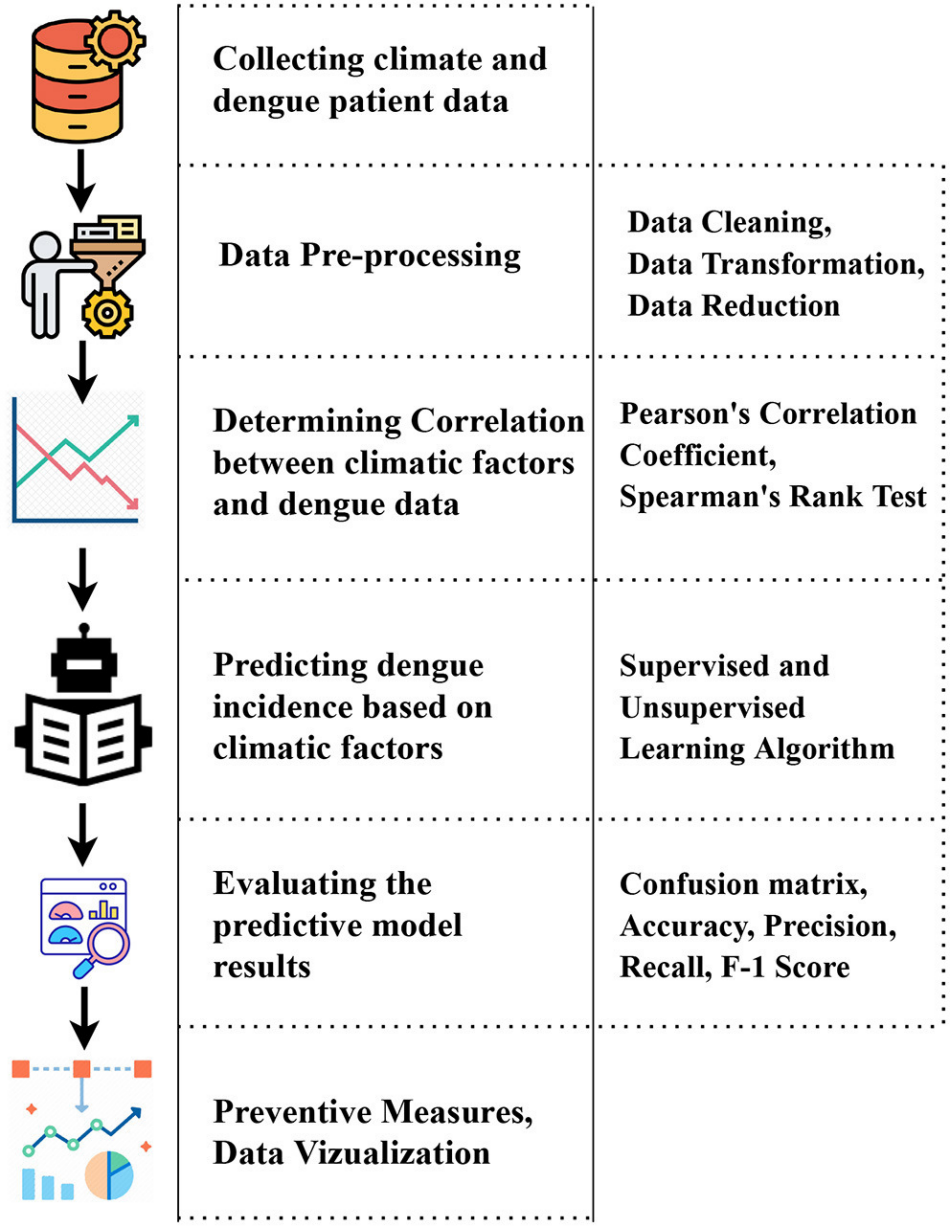
Process of developing a dengue surveillance system.

Various models can predict dengue incidence based on climatic variables (40). Several studies carried out have used different models are described along with the results. Figure 8 depicts the steps and techniques that will required and are essential to develop an early monitoring dengue surveillance system.

#### 3.5.1. Support Vector Regression

SVMs and Support Vector Regression are both based on similar concepts. SVR's main goal is to find the best-fitting line. The hyperplane with the greatest number of points is the best fit line in SVR. In SVR, unlike other regression models, the goal is to fit the best line inside a threshold value. The complexity of SVR's reasonable time grows more than quadratically with the number of samples, making it difficult to scale to datasets with more than a few tens of thousands of samples. Linear SVR is faster than SVR; however, it solely considers the linear kernel. As the cost function ignores samples whose prediction is close to their goal. Based on the evaluation metrics like RMSE and MAE, SVR with linear kernel proved to be quite good at forecasting the number of dengue incidents in Jakarta. Their experiments with various penalty parameters for SVR with linear kernel yielded quite accurate outcomes. When cross-correlation between variables, the linear kernel has a higher prediction accuracy than the radial kernel (41).

#### 3.5.2. Random Forest

Random Forest (42) is an ensemble technique that builds many separate bootstrapped trees from random small subsets of the data using bootstrap aggregation (bagging) (43). As it can handle large numbers of target variables even in complicated interactions, RF is utilized to tackle issues in classification and regression (44). Every tree in the random forest produces a class prediction, and the class with the most votes becomes the prediction of our model. Before training, three hyperparameters of random forest algorithms have to be established. The factors to be considered are the size of nodes, the number of trees and the number of characteristics sampled. This technique is used by Micanaldo et al. (45) in predicting dengue based on various explanatory variables.

#### 3.5.3. Model-Based Recursive Partitioning

The combined effects of the chosen explanatory variables on OI and Dengue fever incidence, Micanaldo et al. (45) used a Model-Based (MOB) recursive partitioning. MOB is similar to CART (classification and regression tree) methods, which recursively partition datasets into subsets depending on independent variables at each step (46). The following steps are repeated iteratively by the MOB algorithm: (1) fit the data to a user-defined linear regression equation; (2) determine if other variables have an impact on model parameters.; (3) if so, using a threshold that results in the most significant changes in the linear model parameters based on the M-fluctuation test, split the model and data into two groups concerning the covariate.; and (4) In each of the subsamples, repeat steps 1–3. Until a specific stop condition is satisfied, the steps are repeated.

#### 3.5.4. Distributed Lag Non-linear Models

Distributed Lag Non-Linear Model is modeling framework for representing links in time series data with non-linear and delayed effects flexibly. The establishment of cross basis, bi-dimensional functional space created by the combination of 2 sets of basis functions that characterize the relationships in the predictor and lag dimensions, respectively, is the cornerstone of this technique.

#### 3.5.5. Generalized Linear Model

Generalized Linear Model is a sophisticated statistical modeling technique that enables us to construct a linear relationship between the answer and the predictors, even though their underlying relationship is not linear. This is possible because of the employment of a link function, which connects the response variable to a linear model. Here, the error distribution of the response variable does not have to be regular.

#### 3.5.6. Generalized Additive Model

Generalized Additive Models are a sort of statistical model. Many nonlinear smooth functions replace the typical linear relationship between the response and targets to represent and capture data non-linearities. These flexible and soft approaches allow us to fit linear models that are either linearly or non-linearly dependent on many predictors to capture nonlinear correlations between response and predictors.

#### 3.5.7. Multilayer Perceptron

A feed-forward ANN called a Multilayer Perceptron is a feed-forward artificial neural network. A hidden layer, an input layer, and an output layer are at least three levels of nodes. For a long time, ANN has been a reliable perceptive classifier for various applications, including medical diagnosis and disease early detection. MLP adapts the classic linear perceptron and employs a supervised learning technique to propagate the network. As a result, it can distinguish facts that cannot be separated. A perceptron forms a linear combination using input weights to produce a single output based on numerous real-valued inputs. They train on a collection of input-output pairs to learn how to express the correlation between inputs and outputs. The model's parameters, or weights and biases, are adjusted throughout training to minimize inaccuracy. Back-propagation is used to change the consequences and preferences about the error, which can be quantified in various ways (47).

#### 3.5.8. K Nearest Neighbor Regression

Scavuzzo et al. (48) used the K-Neighbors Regressor module. Based on k-nearest neighbors, this technique infers a regression. Local interpolation of the training set's targets in the neighborhood is used to forecast the target. Only the first five principal components are used to deconstruct the original data. Four neighbors, Chebyshev metric, brute force and uniform weight, were the tuning settings available. Out of all the tested models, this model gave beast results for modeling the dengue vector population.

#### 3.5.9. Auto-Regressive Integrated Moving Average Model

Xavier et al. (15) chose the ARIMA model, a family of autoregressive moving averages, to describe the relationship among the count of positive dengue cases (dependent variable) and meteorological parameters (explanatory variables). The ARIMA model's primary goal is to directly simulate the autocorrelation in a time series to capture it. Solid underlying mathematics and statistical theory are commonly used to predict time series data, making it easier to construct expected ranges. The ARIMA model is very adaptable, capturing a wide range of patterns. Choosing order and differencing are two fundamental principles in the ARIMA model.

#### 3.5.10. Time Series Poisson Regression Model

Sang et al. (56) to determine the relationship between meteorological conditions and local dengue count, researchers developed this model called Time Series Poisson Regression. According to this model, dengue count was positively related to dengue count in the preceding month, imported cases in the previous month, the minimum temperature during the last month, and accumulative precipitation with 3-month lags.

Table 2 illustrates the detailed representation of latest research papers which has undertaken the study to understand the relation between dengue and meteorological patterns and the need to develop a dengue surveillance system.

**Table 2:** Detailed representations of different models and feature engineering techniques used in different studies.

**References**	**Year**	**Region**	**Techniques**	**Contributions**
Leandro et al. (15)	2021	Brazil	Feature Engineering: ARMAX model	ARMAX model best fits the data used in this study and produced the dengue incidence count with good precision for future.
Joseph et al. (27)	2021	Philippines	Feature Engineering: Pearson's Coefficient Predictive Model: Linear Regression, Exponential Regression	Regression modeling estimated the annual dengue force of infection across urban centers from the age of those with infections and the transmission intensity showed significant spatiotemporal variation.
Daniel et al. (39)	2019	Mexico	Feature Engineering: Multivariate Analysis Predictive Model: Multivariable Logistic Regression Model-MLRM	The connection between dengue and explanatory factors was evaluated via MLRM. A high spatial resolution map was created to highlight the most likely patterns of dengue risk.
Sourabh et al. (38)	2020	India	Feature Engineering: AutoCorrelation Coefficient, Partial Correlation Coefficient and Cross-correlation coefficient Predictive Model: Poisson Distribution Regression Model and Zero Inflated Poisson Regression Model-ZIP	Based on the numerous explanatory factors, the ZIP Model was used to predict the severity of dengue fever in Kolkata.
Oladimeji et al. (34)	2021	Brazil	Feature Engineering: Statistical Analysis Predictive Model: LSTM, RNN	This study used RNN to forecast dengue count and it also uses clustering before modeling which aggregates dengue count with similar temporal patterns.
Micanaldo et al. (45)	2021	Phillipines	Feature Engineering: Cross-correlation Analysis, Variable selection using Random Forest algorithm Predictive Model: Model-based recursive partitioning	MOB recursive partitioning displayed high correlation between dengue transmission and climatic factors and gave accurate predictions.
Sandali et al. (57)	2021	India	Feature Engineering: Data Imputation and Normalization, filling missing values with mean Predictive Model: Artificial Neural Network	A new ANN based multimodal outbreak prediction algorithm is used to predict dengue with the accuracy of 86 percent.
Ivan et al. (41)	2020	Indonesia	Feature Engineering: Cross-correlation, Augmented Dickey Fuller Test Predictive Model: Support Vector Regression	SVR with a linear kernel was applied to climate and dengue incidence data for predicting dengue count and this study also provides a comparative analysis of linear and radial kernel.
Mohd et al. (58)	2019	Malaysia	Feature Engineering: Average Nearest Neighbor (ANN) Predictive Modeling: Spatial Clustering	The hotspot locations were detected using ANN and Kernel density estimation. Based on past data, this study found that it is feasible to estimate dengue risk.
Sabrina et al. (28)	2021	Bangladesh	Feature Engineering: Statistical Analysis Predictive Model: Generalized Additive Model, Generalized Linear Model	The GLM and GAM models were used to show the link between dengue and environmental variables.
Teerawad et al. (32)	2021	Thailand	Feature Engineering: Spearman's Rank Correlation Test Predictive Model: Poisson Regression Model and ARIMA model	Spatial and Temporal modeling of dengue fever transmission was presented and Poisson regression model was used for prediction of dengue based on various climatic factors
Gayan et al. (54)	2018	Sri Lanka	Feature Engineering: Pearson Correlation Coefficient, AutoCorrelation Coefficient, Partial Correlation Coefficient Predictive Model: Time Series Regression Model	The suggested weather-based forecasting algorithm provides high-precision warnings of oncoming dengue outbreaks and epidemics up to one month ahead of time.
Felestin et al. (55)	2021	Malaysia	Feature Engineering: Pearson Correlation Coefficient Predictive Model: Bayes network (BN) models, support vector machine (SVM), RBF tree, decision table and Naive Bayes	The TempeRain factor (TRF), a novel risk factor, was discovered and employed as an input parameter for a dengue epidemic prediction model. The Bayes network produced reliable findings.
Rachel et al. (33)	2021	Brazil	Feature Engineering: Pearson Correlation Test Predictive Model: Spatiotemporal Bayesian Hierarchical Model, Distributed Lag Non-Liner Model	Space-varying, non-linear, and delayed connections between hydrometeorological parameters and dengue incidence are described using coupled spatiotemporal Bayesian hierarchical models with distributed lag non-linear models.
Wu et al. (53)	2021	China	Feature Engineering: Spearman's Correlation Coefficient Predictive Model: Ecological Niche Models(ENM)	ENMs determined the non-random association between dengue count and meteorological factors. Maxnet model was used to predict dengue incidence.

## 4. Discussion

According to the number of people infected, dengue fever is the most common arboviral disease in the world. Understanding the precise relationship between meteorology and dengue transmission is not a simple process because dengue transmission involves dengue viruses, vectors, and susceptible people. Furthermore, forecasting the future of dengue under various climate change scenarios involves a complete understanding of the association between climate and dengue and future climate and other variables. Despite this, a lot of progress has been achieved in this area. Despite the advances achieved in forecasting dengue's future, numerous uncertainties remain (59). To begin with, socio-demographic factors play a significant influence in dengue transmission, and including socio-demographic components into future dengue, projections remains a challenge. Second, in previous studies estimating the future of dengue, the increasing temperature has been commonly employed as a climate change indicator, with rainfall and humidity being under-researched. According to Hales et al., the climate indicator that most correctly predicts the occurrence of dengue fever is vapor pressure, which is a temperature and humidity measurements. However, the relationships between numerous environmental conditions and dengue transmission are complex and sometimes non-linear. Dengue modeling is an essential technique for early detection of dengue outbreaks, assessing risk factors for Severe Dengue, and possibly controlling the disease's vectors. Although much research has been done on these topics, it is critical to understand what areas of dengue modeling still have to be explored to build future research that will significantly reduce disease morbidity rates. The primary goal of this study was to provide an overview of dengue modeling and identify critical issues for future research. This section focuses on the limitations of the studies that have been reviewed. Some research problems or opportunities are described based on those restrictions.

### 4.1. Limitations, Research Challenges and Future Work Directions

#### 4.1.1. Inclusion of Micro Climatic Factors

Researchers have widely used temperature, humidity, and rainfall are macro climatic factors. But to bring in more explanatory variables that can explain the rise in dengue incidence rise can aid in building a better dengue surveillance system (32, 55, 60, 61).

SOLUTION: There are many climatic variables like wind speed, air pressure, vegetation index, and we can add many other microclimatic factors to the model for better dengue incidence

#### 4.1.2. Exploring Different Geographic Locations

Many types of research stated the limitation that how dengue outbreak prediction model developed for one region cannot be used accurately for different areas as dengue incidence may or may not be related to the same climatic factors of one place and due to weather fluctuations and pattern of coastal regions might not give excellent predictive results from the model that was developed for non-coastal regions (55, 62–64).

SOLUTION: Exploring different regions other than that have already been investigated or studying the weather patterns of the country where more minor number studies have been carried out in predicting dengue incidence based on the weather forecast can help in understanding the relationship between different patterns of weather for various regions and dengue rise and eventually would help build a dengue surveillance system.

#### 4.1.3. Data Limitations

Obtaining a dengue dataset that is completely fit for the model and has all characteristics is difficult to find. There are many issues faced by the researchers in this area like i) data being incomplete, ii) data being not available in required time lag like weekly or monthly and for a specific time-period and iii) data that is available only on a macro level like country-level or state-level and not on micro-level like city-level (15, 28, 45, 54, 63, 65).

SOLUTION: Data cleaning can be handled by using various data pre-processing techniques based on the data type. When data is not completed available, taking the average or mean of the available information can be helpful. To obtain data at the micro-level can be a tedious task, but with all the proper procedures, the patient's data can be obtained from the Health Department of the respective region.

#### 4.1.4. Considering Vector Density and Mosquito Larvae Data

The main issue highlighted in most of the studies was the correlation between climatic factors and dengue incidence; whereas those are not the only factors that contribute to increased disease transmission of dengue, the main factor is the vector that carries the transmission (32, 33, 49).

SOLUTION: Considering the vector density, mosquito larvae data or land use would aid in gaining a better understanding of the main factor behind the rise in dengue cases. Considering these parameters would help predict the outbreak better and take preventive measures well in advance.

#### 4.1.5. Taking Into Account the Effect of Vaccination After the Outbreak

The studies carried until now focus on determining the correlation between the climatic factor and dengue incidence and then using weather forecast of coming time to predict the dengue incidence and taking preventive measures. Still, the shortcoming that few researchers found is that there might be a fluctuation in predicting dengue incidence when every infected person might be vaccinated (66–68).

**Figure 9:**
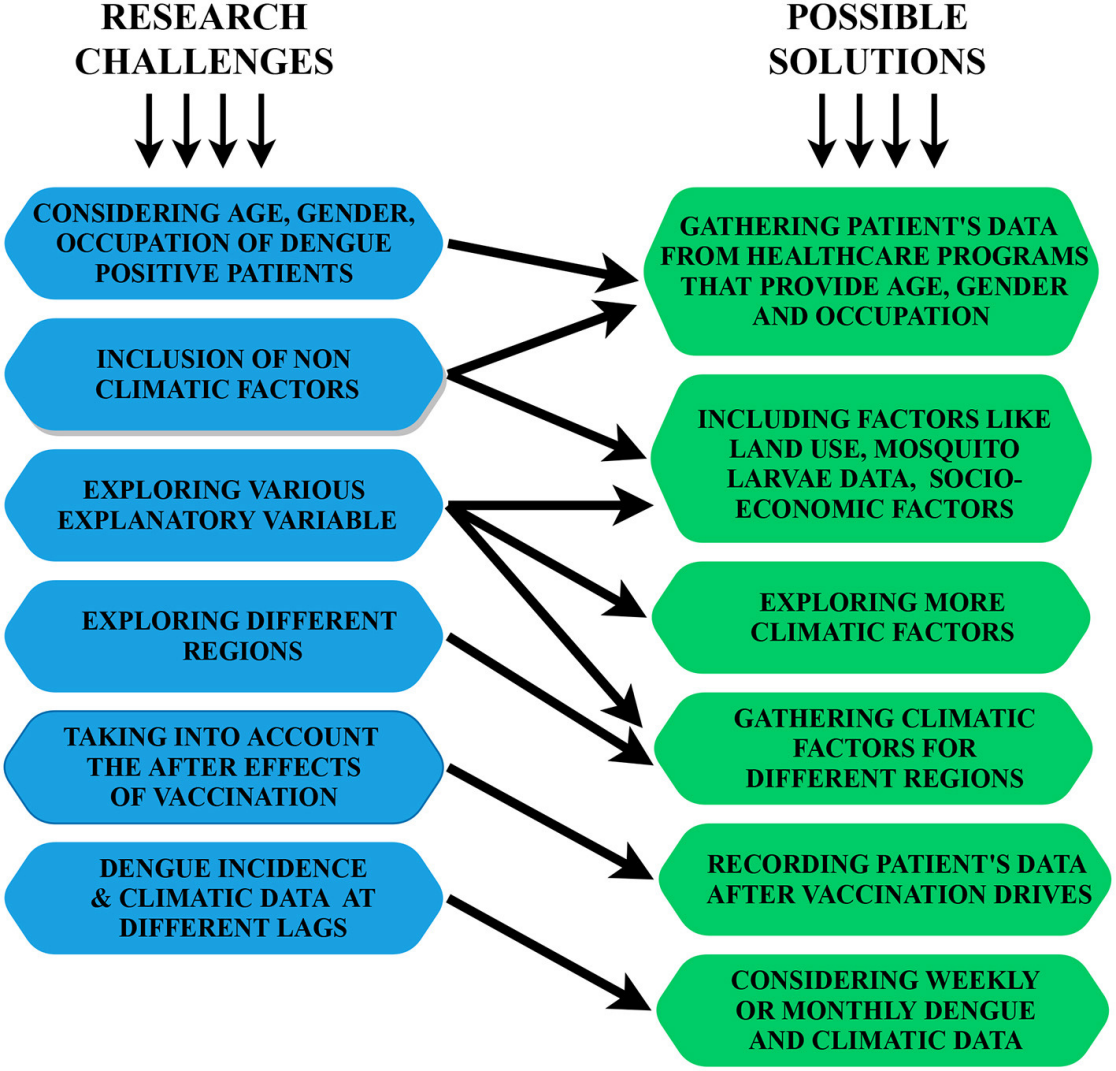
A logical mapping of research challenges and possible solutions.

SOLUTION: As and when the vaccination of patients is done, the data of the patient's immune can be determined and kept a record of. Further, that data can be used whenever the prediction for other outbreaks is being carried out. Figure 9 represents the logical mapping of limitations and their possible solutions.

### 4.2. Contributions

Dengue Surveillance system can aid the health sector in taking preventive measures for dengue outbreaks in advance. Also, it can help to be prepared for the worst of situations and reduce the vulnerability of epidemics. Several research studies have predicted the epidemic using machine learning approaches, including climatic factors. The present review discusses the most common data sources, data pre-processing stages, most common correlation techniques for dengue incidence and climatic factors, machine learning techniques, limitations, and future research directions. Table 3 represents the research challenges of the current study. The contribution of the presented student are:

- The present study gives a detailed insight into the amount of work done in predicting dengue incidence using different machine learning techniques based on various explanatory variables.
- To assist readers, we have discussed several data sources for dengue incidence count and weather variables.
- different approaches for determining the relation between dengue incidence and explanatory variables and various explanatory variables (climatic factors, demographic variables, vulnerable groups).
- Furthermore, we have discussed various dengue modeling techniques for predicting outbreaks.
- This work's main contribution is to outline open research concerns and limitations of various studies.

**Table 3:** Research challenges.

**Reference**	**Year**	**Research challenge**	**Discussion**
Nuraini et al. (64)	2021	Stratifying dengue incidences based on age, gender and occupation	Classifying the population based on different vulnerable groups can help understand which crowd has been affected the most and make future predictions accordingly
Nuraini et al. (64)	2021	Different Regions	Climatic and non-climatic factors vary in different regions. Hence, it is important to understand the geographic and weather patterns to predict dengue accurately for a particular region.
Sriklin et al. (32)	2021	Stratifying dengue incidences based on age, gender and occupation	Non atmospheric data can help understand the factors contributing to dengue and help prevent dengue at an early stage.
Nuraini et al. (64)	2021	Climatic Factors	Rise in dengue incidences became correlated to the temperature and rainfall which would help make dengue outbreak predictions based on weather forecast.
Sriklin et al. (32)	2021	Dataset	Available datasets of dengue incidence were mostly yearly or monthly, hence weekly data could help make accurate predictions. And also the dengue incidences dataset were not accurate and had some missing data regarding patients.

## 5. Conclusion and Future Enhancements

A systematic literature review was conducted on predicting dengue based on climatic change using machine learning techniques. The main objective was to understand the research and studies that have been carried in building a dengue outbreak prediction model. Forty articles were chosen and analyzed to determine the state-of-the-art from many scientific libraries. The results of the review represent that dengue modeling is continuously growing. Logistic Regression, LASSO Regression, and Support Vector Machine approaches were the most commonly used diagnostic models. Because of their ease of implementation and interpretation, they are the most widely used modeling techniques. Although alternative strategies, such as decision trees, are simple to understand, they have a large number of nodes and take a significant amount of mental work to comprehend a specific forecast. These models, on the other hand, are just a set of coefficients, which makes it appealing to learn about the impact of attributes on the predictor variable. Furthermore, continuous independent variables do not have a normal distribution, and continuous and discrete predictors can be used in the regression. In terms of feature types, climate data was the most commonly used in these models. Dengue incidence data of patients is not readily available through APIs, which has been a restriction in many studies and should be considered for future work by forecasting dengue incidence for sensitive groups such as the age and gender of patients. The examined articles had several flaws, including the lack of documentation of the pre-processing procedures employed, the unavailability of patient data, and incomplete data. Following the review of the papers' strengths and shortcomings, future research projects were identified: i) using microclimatic parameters such as wind speed, air pressure, or vegetation index, ii) using demographic and socioeconomic aspects, iii) exploring climatic conditions in different places, iv) using vector density data, and v) taking vaccination drives into account when building a prediction model. Forecasting the future of dengue fever in the context of climate change can assist governments and public health professionals in implementing timely and preventative steps to protect people from dengue in the future. To sum up, climate change is creating an alarming situation affecting many sectors. Dengue has been a reason of concern for a long time. Hence, monitoring the fluctuations of weather patterns can aid in creating a dengue surveillance system that would be of great help to the health sector in taking preventive measures well in advance.

## Data Availability Statement

The original contributions presented in the study are included in the article/supplementary material, further inquiries can be directed to the corresponding author.

## Author Contributions

SPat, SPan, and DB: conceptualization. SB, QI, and SI: funding acquisition. DB and SPat: investigation and methodology, writing of the original draft, and data curation. SB: project administration and visualization. QI and SI: resources and supervision. SPan and SB: writing of the review and editing. SPan: validation. All authors contributed to the article and approved the submitted version.

## Funding

This work was supported by the Deanship of Scientific Research, Vice Presidency for Graduate Studies and Scientific Research, King Faisal University, Saudi Arabia (Project no. GRANT 268).

## Conflict of Interest

The authors declare that the research was conducted in the absence of any commercial or financial relationships that could be construed as a potential conflict of interest.

## Publisher's Note

All claims expressed in this article are solely those of the authors and do not necessarily represent those of their affiliated organizations, or those of the publisher, the editors and the reviewers. Any product that may be evaluated in this article, or claim that may be made by its manufacturer, is not guaranteed or endorsed by the publisher.
